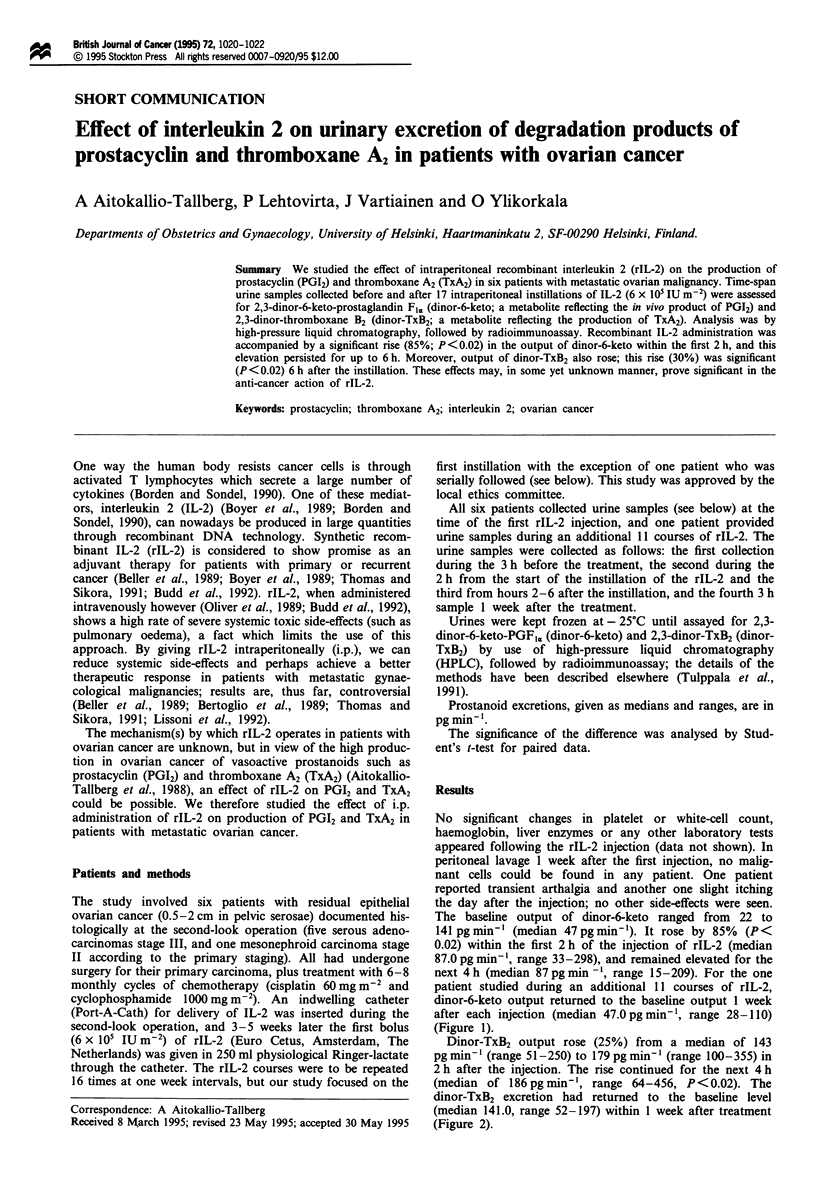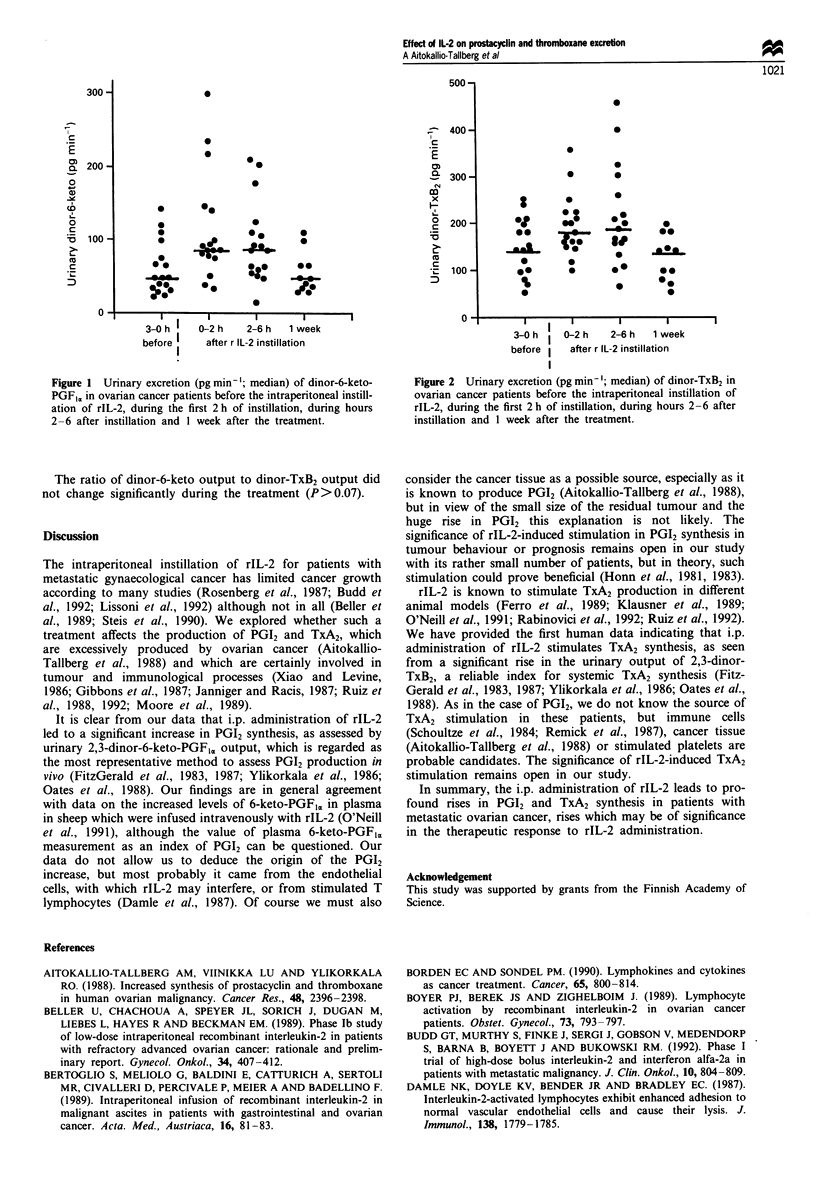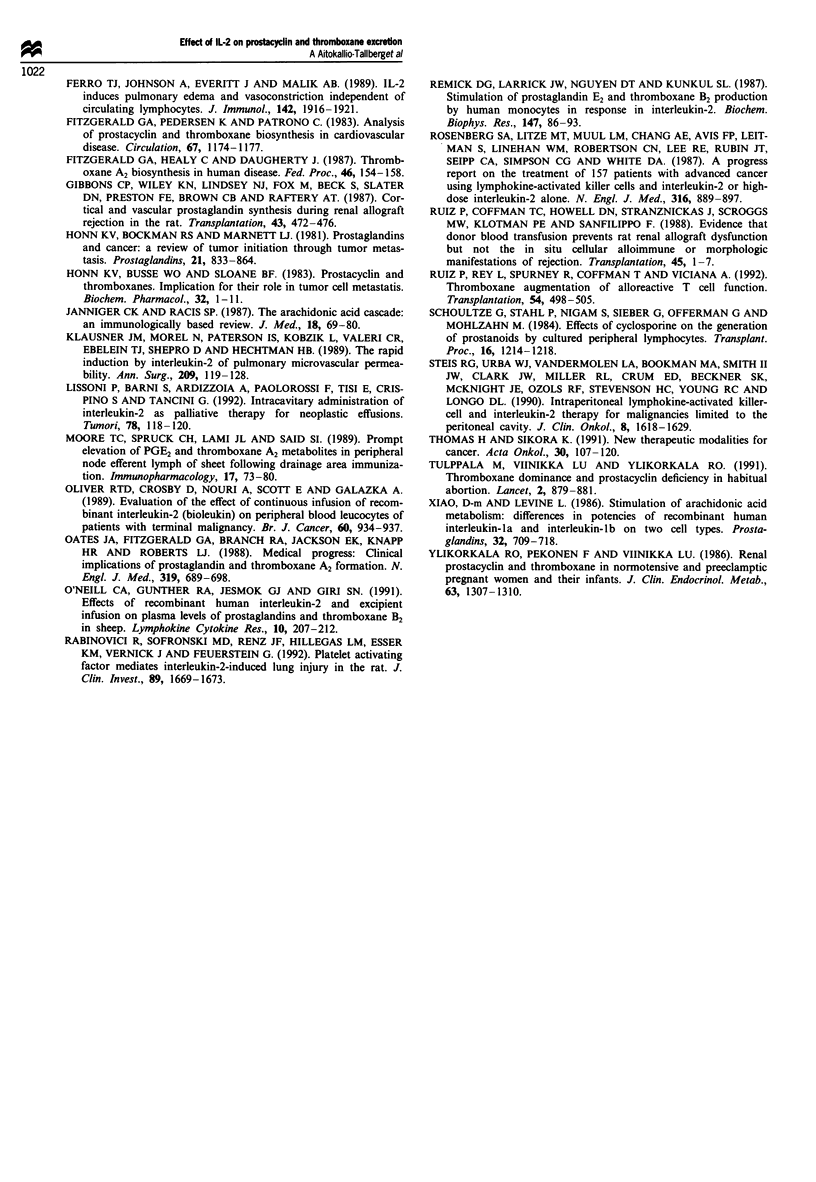# Effect of interleukin 2 on urinary excretion of degradation products of prostacyclin and thromboxane A2 in patients with ovarian cancer.

**DOI:** 10.1038/bjc.1995.454

**Published:** 1995-10

**Authors:** A. Aitokallio-Tallberg, P. Lehtovirta, J. Vartiainen, O. Ylikorkala

**Affiliations:** Department of Obstetrics and Gynaecology, University of Helsinki, Finland.

## Abstract

We studied the effect of intraperitoneal recombinant interleukin 2 (rIL-2) on the production of prostacyclin (PGI2) and thromboxane A2 (TxA2) in six patients with metastatic ovarian malignancy. Time-span urine samples collected before and after 17 intraperitoneal instillations of IL-2 (6 x 10(5) IU m-2) were assessed for 2,3-dinor-6-keto-prostaglandin F1 alpha (dinor-6-keto; a metabolite reflecting the in vivo product of PGI2) and 2,3-dinor-thromboxane B2 (dinor-TxB2; a metabolite reflecting the production of TxA2). Analysis was by high-pressure liquid chromatography, followed by radioimmunoassay. Recombinant IL-2 administration was accompanied by a significant rise (85%; P < 0.02) in the output of dinor-6-keto within the first 2 h, and this elevation persisted for up to 6 h. Moreover, output of dinor-TxB2 also rose; this rise (30%) was significant (P < 0.02) 6 h after the instillation. These effects may, in some yet unknown manner, prove significant in the anti-cancer action of rIL-2.


British Journal of Cancer (1995) 72, 1020-1022
© 1995 Stockton Press All rights reserved 0007-0920/95 $12.00

SHORT COMMUNICATION

### Effect of interleukin 2 on urinary excretion of degradation products of prostacyclin and thromboxane $A_2$ in patients with ovarian cancer

A Aitokallio-Tallberg, P Lehtovirta, J Vartiainen and O Ylikorkala

*Departments of Obstetrics and Gynaecology, University of Helsinki, Haartmaninkatu 2, SF-00290 Helsinki, Finland.*

**Summary** We studied the effect of intraperitoneal recombinant interleukin 2 (rIL-2) on the production of prostacyclin ($PGI_2$) and thromboxane $A_2$ ($TxA_2$) in six patients with metastatic ovarian malignancy. Time-span urine samples collected before and after 17 intraperitoneal instillations of IL-2 ($6 \times 10^5$ IU $m^{-2}$) were assessed for 2,3-dinor-6-keto-prostaglandin $F_{1\alpha}$ (dinor-6-keto; a metabolite reflecting the *in vivo* product of $PGI_2$) and 2,3-dinor-thromboxane $B_2$ (dinor-$TxB_2$; a metabolite reflecting the production of $TxA_2$). Analysis was by high-pressure liquid chromatography, followed by radioimmunoassay. Recombinant IL-2 administration was accompanied by a significant rise (85%; $P<0.02$) in the output of dinor-6-keto within the first 2 h, and this elevation persisted for up to 6 h. Moreover, output of dinor-$TxB_2$ also rose; this rise (30%) was significant ($P<0.02$) 6 h after the instillation. These effects may, in some yet unknown manner, prove significant in the anti-cancer action of rIL-2.

**Keywords:** prostacyclin; thromboxane $A_2$; interleukin 2; ovarian cancer

One way the human body resists cancer cells is through activated T lymphocytes which secrete a large number of cytokines (Borden and Sondel, 1990). One of these mediators, interleukin 2 (IL-2) (Boyer *et al.*, 1989; Borden and Sondel, 1990), can nowadays be produced in large quantities through recombinant DNA technology. Synthetic recombinant IL-2 (rIL-2) is considered to show promise as an adjuvant therapy for patients with primary or recurrent cancer (Beller *et al.*, 1989; Boyer *et al.*, 1989; Thomas and Sikora, 1991; Budd *et al.*, 1992). rIL-2, when administered intravenously however (Oliver *et al.*, 1989; Budd *et al.*, 1992), shows a high rate of severe systemic toxic side-effects (such as pulmonary oedema), a fact which limits the use of this approach. By giving rIL-2 intraperitoneally (i.p.), we can reduce systemic side-effects and perhaps achieve a better therapeutic response in patients with metastatic gynaecological malignancies; results are, thus far, controversial (Beller *et al.*, 1989; Bertoglio *et al.*, 1989; Thomas and Sikora, 1991; Lissoni *et al.*, 1992).

The mechanism(s) by which rIL-2 operates in patients with ovarian cancer are unknown, but in view of the high production in ovarian cancer of vasoactive prostanoids such as prostacyclin ($PGI_2$) and thromboxane $A_2$ ($TxA_2$) (Aitokallio-Tallberg *et al.*, 1988), an effect of rIL-2 on $PGI_2$ and $TxA_2$ could be possible. We therefore studied the effect of i.p. administration of rIL-2 on production of $PGI_2$ and $TxA_2$ in patients with metastatic ovarian cancer.

### Patients and methods

The study involved six patients with residual epithelial ovarian cancer (0.5–2 cm in pelvic serosae) documented histologically at the second-look operation (five serous adenocarcinomas stage III, and one mesonephroid carcinoma stage II according to the primary staging). All had undergone surgery for their primary carcinoma, plus treatment with 6–8 monthly cycles of chemotherapy (cisplatin 60 mg $m^{-2}$ and cyclophosphamide 1000 mg $m^{-2}$). An indwelling catheter (Port-A-Cath) for delivery of IL-2 was inserted during the second-look operation, and 3–5 weeks later the first bolus ($6 \times 10^5$ IU $m^{-2}$) of rIL-2 (Euro Cetus, Amsterdam, The Netherlands) was given in 250 ml physiological Ringer-lactate through the catheter. The rIL-2 courses were to be repeated 16 times at one week intervals, but our study focused on the first instillation with the exception of one patient who was serially followed (see below). This study was approved by the local ethics committee.

All six patients collected urine samples (see below) at the time of the first rIL-2 injection, and one patient provided urine samples during an additional 11 courses of rIL-2. The urine samples were collected as follows: the first collection during the 3 h before the treatment, the second during the 2 h from the start of the instillation of the rIL-2 and the third from hours 2–6 after the instillation, and the fourth 3 h sample 1 week after the treatment.

Urines were kept frozen at $-25°C$ until assayed for 2,3-dinor-6-keto-$PGF_{1\alpha}$ (dinor-6-keto) and 2,3-dinor-$TxB_2$ (dinor-$TxB_2$) by use of high-pressure liquid chromatography (HPLC), followed by radioimmunoassay; the details of the methods have been described elsewhere (Tulppala *et al.*, 1991).

Prostanoid excretions, given as medians and ranges, are in pg $min^{-1}$.

The significance of the difference was analysed by Student's *t*-test for paired data.

### Results

No significant changes in platelet or white-cell count, haemoglobin, liver enzymes or any other laboratory tests appeared following the rIL-2 injection (data not shown). In peritoneal lavage 1 week after the first injection, no malignant cells could be found in any patient. One patient reported transient arthalgia and another one slight itching the day after the injection; no other side-effects were seen. The baseline output of dinor-6-keto ranged from 22 to 141 pg $min^{-1}$ (median 47 pg $min^{-1}$). It rose by 85% ($P<0.02$) within the first 2 h of the injection of rIL-2 (median 87.0 pg $min^{-1}$, range 33–298), and remained elevated for the next 4 h (median 87 pg $min^{-1}$, range 15–209). For the one patient studied during an additional 11 courses of rIL-2, dinor-6-keto output returned to the baseline output 1 week after each injection (median 47.0 pg $min^{-1}$, range 28–110) (Figure 1).

Dinor-$TxB_2$ output rose (25%) from a median of 143 pg $min^{-1}$ (range 51–250) to 179 pg $min^{-1}$ (range 100–355) in 2 h after the injection. The rise continued for the next 4 h (median of 186 pg $min^{-1}$, range 64–456, $P<0.02$). The dinor-$TxB_2$ excretion had returned to the baseline level (median 141.0, range 52–197) within 1 week after treatment (Figure 2).

Correspondence: A Aitokallio-Tallberg
Received 8 March 1995; revised 23 May 1995; accepted 30 May 1995

**Figure 1** Urinary excretion (pg $min^{-1}$; median) of dinor-6-keto-$PGF_{1\alpha}$ in ovarian cancer patients before the intraperitoneal instillation of rIL-2, during the first 2 h of instillation, during hours 2–6 after instillation and 1 week after the treatment.

**Figure 2** Urinary excretion (pg $min^{-1}$; median) of dinor-$TxB_2$ in ovarian cancer patients before the intraperitoneal instillation of rIL-2, during the first 2 h of instillation, during hours 2–6 after instillation and 1 week after the treatment.

The ratio of dinor-6-keto output to dinor-$TxB_2$ output did not change significantly during the treatment ($P > 0.07$).

### Discussion

The intraperitoneal instillation of rIL-2 for patients with metastatic gynaecological cancer has limited cancer growth according to many studies (Rosenberg *et al.*, 1987; Budd *et al.*, 1992; Lissoni *et al.*, 1992) although not in all (Beller *et al.*, 1989; Steis *et al.*, 1990). We explored whether such a treatment affects the production of $PGI_2$ and $TxA_2$, which are excessively produced by ovarian cancer (Aitokallio-Tallberg *et al.*, 1988) and which are certainly involved in tumour and immunological processes (Xiao and Levine, 1986; Gibbons *et al.*, 1987; Janniger and Racis, 1987; Ruiz *et al.*, 1988, 1992; Moore *et al.*, 1989).

It is clear from our data that i.p. administration of rIL-2 led to a significant increase in $PGI_2$ synthesis, as assessed by urinary 2,3-dinor-6-keto-$PGF_{1\alpha}$ output, which is regarded as the most representative method to assess $PGI_2$ production *in vivo* (FitzGerald *et al.*, 1983, 1987; Ylikorkala *et al.*, 1986; Oates *et al.*, 1988). Our findings are in general agreement with data on the increased levels of 6-keto-$PGF_{1\alpha}$ in plasma in sheep which were infused intravenously with rIL-2 (O'Neill *et al.*, 1991), although the value of plasma 6-keto-$PGF_{1\alpha}$ measurement as an index of $PGI_2$ can be questioned. Our data do not allow us to deduce the origin of the $PGI_2$ increase, but most probably it came from the endothelial cells, with which rIL-2 may interfere, or from stimulated T lymphocytes (Damle *et al.*, 1987). Of course we must also consider the cancer tissue as a possible source, especially as it is known to produce $PGI_2$ (Aitokallio-Tallberg *et al.*, 1988), but in view of the small size of the residual tumour and the huge rise in $PGI_2$ this explanation is not likely. The significance of rIL-2-induced stimulation in $PGI_2$ synthesis in tumour behaviour or prognosis remains open in our study with its rather small number of patients, but in theory, such stimulation could prove beneficial (Honn *et al.*, 1981, 1983).

rIL-2 is known to stimulate $TxA_2$ production in different animal models (Ferro *et al.*, 1989; Klausner *et al.*, 1989; O'Neill *et al.*, 1991; Rabinovici *et al.*, 1992; Ruiz *et al.*, 1992). We have provided the first human data indicating that i.p. administration of rIL-2 stimulates $TxA_2$ synthesis, as seen from a significant rise in the urinary output of 2,3-dinor-$TxB_2$, a reliable index for systemic $TxA_2$ synthesis (Fitz-Gerald *et al.*, 1983, 1987; Ylikorkala *et al.*, 1986; Oates *et al.*, 1988). As in the case of $PGI_2$, we do not know the source of $TxA_2$ stimulation in these patients, but immune cells (Schoultze *et al.*, 1984; Remick *et al.*, 1987), cancer tissue (Aitokallio-Tallberg *et al.*, 1988) or stimulated platelets are probable candidates. The significance of rIL-2-induced $TxA_2$ stimulation remains open in our study.

In summary, the i.p. administration of rIL-2 leads to profound rises in $PGI_2$ and $TxA_2$ synthesis in patients with metastatic ovarian cancer, rises which may be of significance in the therapeutic response to rIL-2 administration.

**Acknowledgement**
This study was supported by grants from the Finnish Academy of Science.

### References

AITOKALLIO-TALLBERG AM, VIINIKKA LU AND YLIKORKALA RO. (1988). Increased synthesis of prostacyclin and thromboxane in human ovarian malignancy. *Cancer Res.*, **48**, 2396–2398.

BELLER U, CHACHOUA A, SPEYER JL, SORICH J, DUGAN M, LIEBES L, HAYES R AND BECKMAN EM. (1989). Phase Ib study of low-dose intraperitoneal recombinant interleukin-2 in patients with refractory advanced ovarian cancer: rationale and preliminary report. *Gynecol. Onkol.*, **34**, 407–412.

BERTOGLIO S, MELIOLO G, BALDINI E, CATTURICH A, SERTOLI MR, CIVALLERI D, PERCIVALE P, MEIER A AND BADELLINO F. (1989). Intraperitoneal infusion of recombinant interleukin-2 in malignant ascites in patients with gastrointestinal and ovarian cancer. *Acta. Med., Austriaca*, **16**, 81–83.

BORDEN EC AND SONDEL PM. (1990). Lymphokines and cytokines as cancer treatment. *Cancer*, **65**, 800–814.

BOYER PJ, BEREK JS AND ZIGHELBOIM J. (1989). Lymphocyte activation by recombinant interleukin-2 in ovarian cancer patients. *Obstet. Gynecol.*, **73**, 793–797.

BUDD GT, MURTHY S, FINKE J, SERGI J, GOBSON V, MEDENDORP S, BARNA B, BOYETT J AND BUKOWSKI RM. (1992). Phase I trial of high-dose bolus interleukin-2 and interferon alfa-2a in patients with metastatic malignancy. *J. Clin. Onkol.*, **10**, 804–809.

DAMLE NK, DOYLE KV, BENDER JR AND BRADLEY EC. (1987). Interleukin-2-activated lymphocytes exhibit enhanced adhesion to normal vascular endothelial cells and cause their lysis. *J. Immunol.*, **138**, 1779–1785.

FERRO TJ, JOHNSON A, EVERITT J AND MALIK AB. (1989). IL-2 induces pulmonary edema and vasoconstriction independent of circulating lymphocytes. *J. Immunol.*, **142**, 1916–1921.

FITZGERALD GA, PEDERSEN K AND PATRONO C. (1983). Analysis of prostacyclin and thromboxane biosynthesis in cardiovascular disease. *Circulation*, **67**, 1174–1177.

FITZGERALD GA, HEALY C AND DAUGHERTY J. (1987). Thromboxane $A_2$ biosynthesis in human disease. *Fed. Proc.*, **46**, 154–158.

GIBBONS CP, WILEY KN, LINDSEY NJ, FOX M, BECK S, SLATER DN, PRESTON FE, BROWN CB AND RAFTERY AT. (1987). Cortical and vascular prostaglandin synthesis during renal allograft rejection in the rat. *Transplantation*, **43**, 472–476.

HONN KV, BOCKMAN RS AND MARNETT LJ. (1981). Prostaglandins and cancer: a review of tumor initiation through tumor metastasis. *Prostaglandins*, **21**, 833–864.

HONN KV, BUSSE WO AND SLOANE BF. (1983). Prostacyclin and thromboxanes. Implication for their role in tumor cell metastatis. *Biochem. Pharmacol.*, **32**, 1–11.

JANNIGER CK AND RACIS SP. (1987). The arachidonic acid cascade: an immunologically based review. *J. Med.*, **18**, 69–80.

KLAUSNER JM, MOREL N, PATERSON IS, KOBZIK L, VALERI CR, EBELEIN TJ, SHEPRO D AND HECHTMAN HB. (1989). The rapid induction by interleukin-2 of pulmonary microvascular permeability. *Ann. Surg.*, **209**, 119–128.

LISSONI P, BARNI S, ARDIZZOIA A, PAOLOROSSI F, TISI E, CRISPINO S AND TANCINI G. (1992). Intracavitary administration of interleukin-2 as palliative therapy for neoplastic effusions. *Tumori*, **78**, 118–120.

MOORE TC, SPRUCK CH, LAMI JL AND SAID SI. (1989). Prompt elevation of $PGE_2$ and thromboxane $A_2$ metabolites in peripheral node efferent lymph of sheet following drainage area immunization. *Immunopharmacology*, **17**, 73–80.

OLIVER RTD, CROSBY D, NOURI A, SCOTT E AND GALAZKA A. (1989). Evaluation of the effect of continuous infusion of recombinant interleukin-2 (bioleukin) on peripheral blood leucocytes of patients with terminal malignancy. *Br. J. Cancer*, **60**, 934–937.

OATES JA, FITZGERALD GA, BRANCH RA, JACKSON EK, KNAPP HR AND ROBERTS LJ. (1988). Medical progress: Clinical implications of prostaglandin and thromboxane $A_2$ formation. *N. Engl. J. Med.*, **319**, 689–698.

O'NEILL CA, GUNTHER RA, JESMOK GJ AND GIRI SN. (1991). Effects of recombinant human interleukin-2 and excipient infusion on plasma levels of prostaglandins and thromboxane $B_2$ in sheep. *Lymphokine Cytokine Res.*, **10**, 207–212.

RABINOVICI R, SOFRONSKI MD, RENZ JF, HILLEGAS LM, ESSER KM, VERNICK J AND FEUERSTEIN G. (1992). Platelet activating factor mediates interleukin-2-induced lung injury in the rat. *J. Clin. Invest.*, **89**, 1669–1673.

REMICK DG, LARRICK JW, NGUYEN DT AND KUNKUL SL. (1987). Stimulation of prostaglandin $E_2$ and thromboxane $B_2$ production by human monocytes in response in interleukin-2. *Biochem. Biophys. Res.*, **147**, 86–93.

ROSENBERG SA, LITZE MT, MUUL LM, CHANG AE, AVIS FP, LEITMAN S, LINEHAN WM, ROBERTSON CN, LEE RE, RUBIN JT, SEIPP CA, SIMPSON CG AND WHITE DA. (1987). A progress report on the treatment of 157 patients with advanced cancer using lymphokine-activated killer cells and interleukin-2 or high-dose interleukin-2 alone. *N. Engl. J. Med.*, **316**, 889–897.

RUIZ P, COFFMAN TC, HOWELL DN, STRANZNICKAS J, SCROGGS MW, KLOTMAN PE AND SANFILIPPO F. (1988). Evidence that donor blood transfusion prevents rat renal allograft dysfunction but not the in situ cellular alloimmune or morphologic manifestations of rejection. *Transplantation*, **45**, 1–7.

RUIZ P, REY L, SPURNEY R, COFFMAN T AND VICIANA A. (1992). Thromboxane augmentation of alloreactive T cell function. *Transplantation*, **54**, 498–505.

SCHOULTZE G, STAHL P, NIGAM S, SIEBER G, OFFERMAN G AND MOHLZAHN M. (1984). Effects of cyclosporine on the generation of prostanoids by cultured peripheral lymphocytes. *Transplant. Proc.*, **16**, 1214–1218.

STEIS RG, URBA WJ, VANDERMOLEN LA, BOOKMAN MA, SMITH II JW, CLARK JW, MILLER RL, CRUM ED, BECKNER SK, MCKNIGHT JE, OZOLS RF, STEVENSON HC, YOUNG RC AND LONGO DL. (1990). Intraperitoneal lymphokine-activated killer-cell and interleukin-2 therapy for malignancies limited to the peritoneal cavity. *J. Clin. Onkol.*, **8**, 1618–1629.

THOMAS H AND SIKORA K. (1991). New therapeutic modalities for cancer. *Acta Onkol.*, **30**, 107–120.

TULPPALA M, VIINIKKA LU AND YLIKORKALA RO. (1991). Thromboxane dominance and prostacyclin deficiency in habitual abortion. *Lancet*, **2**, 879–881.

XIAO, D-m AND LEVINE L. (1986). Stimulation of arachidonic acid metabolism: differences in potencies of recombinant human interleukin-1a and interleukin-1b on two cell types. *Prostaglandins*, **32**, 709–718.

YLIKORKALA RO, PEKONEN F AND VIINIKKA LU. (1986). Renal prostacyclin and thromboxane in normotensive and preeclamptic pregnant women and their infants. *J. Clin. Endocrinol. Metab.*, **63**, 1307–1310.